# Huntingtin gene evolution in Chordata and its peculiar features in the ascidian *Ciona *genus

**DOI:** 10.1186/1471-2164-7-288

**Published:** 2006-11-08

**Authors:** Carmela Gissi, Graziano Pesole, Elena Cattaneo, Marzia Tartari

**Affiliations:** 1 Dipartimento di Scienze Biomolecolari e Biotecnologie, Università di Milano, Milano, Italy; 2 Dipartimento di Biochimica e Biologia Molecolare, Università di Bari, Bari, Italy; 3 Department of Pharmacological Sciences and Center of Excellence on Neurodegenerative Diseases, University of Milano, Milano, Italy

## Abstract

**Background:**

To gain insight into the evolutionary features of the huntingtin (htt) gene in Chordata, we have sequenced and characterized the full-length htt mRNA in the ascidian *Ciona intestinalis*, a basal chordate emerging as new invertebrate model organism. Moreover, taking advantage of the availability of genomic and EST sequences, the htt gene structure of a number of chordate species, including the cogeneric ascidian *Ciona savignyi*, and the vertebrates *Xenopus *and *Gallus *was reconstructed.

**Results:**

The *C. intestinalis *htt transcript exhibits some peculiar features, such as spliced leader trans-splicing in the 98 nt-long 5' untranslated region (UTR), an alternative splicing in the coding region, eight alternative polyadenylation sites, and no similarities of both 5' and 3'UTRs compared to homologs of the cogeneric *C. savignyi*. The predicted protein is 2946 amino acids long, shorter than its vertebrate homologs, and lacks the polyQ and the polyP stretches found in the the N-terminal regions of mammalian homologs. The exon-intron organization of the htt gene is almost identical among vertebrates, and significantly conserved between *Ciona *and vertebrates, allowing us to hypothesize an ancestral chordate gene consisting of at least 40 coding exons.

**Conclusion:**

During chordate diversification, events of gain/loss, sliding, phase changes, and expansion of introns occurred in both vertebrate and ascidian lineages predominantly in the 5'-half of the htt gene, where there is also evidence of lineage-specific evolutionary dynamics in vertebrates. On the contrary, the 3'-half of the gene is highly conserved in all chordates at the level of both gene structure and protein sequence. Between the two *Ciona *species, a fast evolutionary rate and/or an early divergence time is suggested by the absence of significant similarity between UTRs, protein divergence comparable to that observed between mammals and fishes, and different distribution of repetitive elements.

## Background

Huntingtin is a large protein (> 3000 amino acids long in vertebrates), characterized in humans by the presence of an amino-terminal polymorphic polyglutamine (polyQ) tract whose aberrant expansion causes Huntington's Disease (HD), a progressive neurodegenerative disease accompanied by neuronal dysfunction and cell loss in the brain [1]. HD is dominantly inherited, and this observation, together with a number of important experimental results, point to the polyQ tract in the mutant protein as disease-triggering [2]. More recently, it has been proposed that aspects of the disease might be caused by reduced activity of the normal huntingtin protein, indeed a number of biological investigations point to individual protective activities of huntingtin in brain cells [3]. The protein is expressed ubiquitously in humans and rodents, with the highest levels in the neurons of the CNS [4]. Its widespread intracellular localisation does not facilitate the definition of its physiological function. In addition, the large dimension of the protein makes its purification extremely problematic and its three dimensional structure remains unsolved. Because of these difficulties, the study of huntingtin function(s) has largely progressed through gene deletion and gene addition studies that have demonstrated that huntingtin is essential for embryonic development [3]. In addition, huntingtin is important for the survival of brain neurons where it also controls BDNF (brain-derived neurotrophic factor) production and delivery [5-7], axonal transport [8,9] and neuronal gene transcription [3].

The huntingtin gene or transcript has been characterized in a number of vertebrate and invertebrate species [10-17], and it seems to be absent in yeast and lower eukaryotes [16]. Current data demonstrate that huntingtin is a single copy gene – highly conserved in vertebrates but poorly conserved or absent in invertebrates [16]. Indeed, in *Drosophila melanogaster *huntingtin shows only five regions, accounting for 36% of the whole protein, with high similarity to vertebrate homologs [16], while the gene has not been detected in the nematode *Caenorhabditis elegans*. In invertebrate deuterostomes (the tunicate *Halocynthia roretzi *and two echinodems), only cDNA clones corresponding approximately to the last three hundred amino acids of the protein have been identified and used to investigate the expression pattern, suggesting that neural expression emerged as novel feature in the phylum of chordates, and was absent in primitive deuterostomes [17]. As further peculiarity, huntingtin does not present regions of similarity to other proteins or genes. Only a conserved secondary structure domain has been identified as shared with other proteins, indeed huntingtin is notable for contributing to the definition of HEAT repeats (Huntingtin, Elongation factor 3, protein phosphatase 2A, TOR1), a 37–43 amino acid motif consisting of two alpha helices forming a helical hairpin [18-20]. The HEAT repeats are normally present in tandem arrays or clusters and are indicative of the ability of the protein to mediate protein-protein interactions [21,22], however they provide only limited clues to the protein function.

Current data thus provide little help in speculations as to the origin and evolution of this large protein, as well as of its Q-rich domain. All comparative studies carried out so far have been focused on the protein, with few studies analyzing the gene structure in single species. In the case of a highly conserved protein such as huntingtin, a detailed comparative study of the exon-intron structure of the gene and its evolution could shed light on the evolution of this gene across chordates and help to identify the regions of the gene (and consequently of the protein) evolving under different functional constraints. The current availability of complete genome sequences for a number of organisms offers the possibility to identify and annotate this large gene and to study its evolutionary dynamics in a large taxonomic sample. In this study we focus our attention on the basal chordate *Ciona intestinalis *(Tunicata, Ascidiacea). This model organism is of fundamental importance in evolutionary studies, because it has the advantage of being a chordate-invertebrate: as a chordate, this species shows a body plan (at least in the tadpole larval stage) and embryonic development very similar to those of vertebrates [23] but, as an invertebrate, it exhibits enough genetic divergence from vertebrates to allow more incisive evolutionary and comparative analyses at protein level. Thus, the large evolutionary distance separating tunicates and vertebrates (about 520 million years) [24] could allow the identification of a huntingtin "signature" related to the ancestral chordate function of the gene/protein.

In this study we report the characterization of the *Ciona intestinalis *huntingtin full-length transcript, including the 5' and 3' untranslated regions (UTRs), and the definition of its gene structure using available *C. intestinalis *genomic sequences. We have thus carried out a comprehensive comparative analysis of the exon-intron structure of the huntingtin gene in chordates, that is between two cogeneric ascidians *Ciona intestinalis *and *Ciona savignyi *and eight vertebrate species, including *Xenopus *and *Gallus *– whose gene structures were here accurately predicted from genomic sequences.

## Results

### The *Ciona *huntingtin transcript

The full-length transcript of the *C. intestinalis *huntingtin (htt) shows several peculiar features. It ranges from 8919 to 9957 nucleotides (nt) in length, depending on the usage of an alternative splicing site in the coding region and of the polyadenylation site.

**Figure 1:**
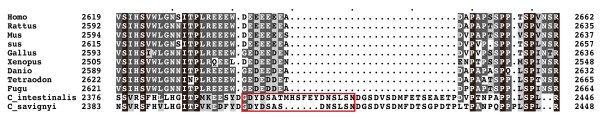
**Amino acid alignment of the huntingtin region corresponding to exon 50–51 of *Ciona***. The red box indicates the region absent in the alternatively spliced isoform. Identical, similar and conserved positions are reported with different backgrounds.

The longest protein-coding region (CDS), from the first AUG to the UGA stop codon, is 8841 bp long. A competing 5' splice site in exon 50 allows the synthesis of an alternative transcript lacking 57 nt (19 amino acids) in the C-terminal portion of the encoded protein (Figure 1). In the first exon of *C. intestinalis *there are no in-frame upstream or downstream AUG with respect to the inferred initiator, whereas in exon 1 of vertebrates there are one or two additional Met codons, depending on the species: in position 4 in non-mammalian species, and in position 8 in all vertebrates. The start codon of *C. intestinalis *htt is unambiguously homologous to the Met at position 4, common only to non-mammalian vertebrates (see Additional file 1). The same situation is found in *C. savignyi*.

The complete 5' untranslated region (5'UTR) was determined by 5'RACE and is 98 bp long. As revealed by comparison with the genomic sequence, most of the sequence is encoded by the exon that contains the translation start codon. Surprisingly, the first bases of the 5'UTR were found neither in the first exon, nor in the genomic region 10 kb upstream the first exon, excluding the existence of an upstream micro-exon. On the contrary, this sequence perfectly matches the 16-bp long sequence recognized as spliced leader (SL) in *C. intestinalis *[25], pointing to an event of trans-splicing in the htt mRNA maturation. The presence of a SL sequence in the mature htt transcript of *Ciona *prevents both the identification of the transcription start site and the "in silico" characterization of the promoter region.

Although quite short, the 5'UTR contains two upstream ORFs (uORF) that are out-of frame with respect the to translation start: the first uORF is located at position 49 and is immediately followed by a UGA stop codon; the second uORF starts at position 68 and is 15 nt long (amino acid sequence MLSFI, stop codon UAG). In *C. savignyi*, no sequences with similarity to the *C. intestinalis *5'UTR were found in the 10 kbp upstream of the first coding exon. Assuming that as in *C. savignyi *the sequence 98 bp-long immediately upstream the translation start codon constitutes the 5'UTR, two out-of frame uORFs (60 and 27 nt long) were found in this putative 5'UTR. Moreover, all uORFs exhibit a start codon context different from the Kozak's consensus [26] and there is no similarity between the uORFs of *C. intestinalis *and *C. savignyi*.

The start codon context is aaacauaAUGgaa in *C. intestinalis*, and cauugcgAUGgaa in *C. savignyi *(assuming the 5'UTR as reported before), thus the purine 3 nt upstream of the start codon, and the G immediately after the start codon are conserved, suggesting a strong context of translation initiation [26].

Seven alternative polyadenylation (polyA) sites were experimentally identified by 3'RACE, and an additional site was found in two EST clones, making a total of eight alternative polyA sites (Table 1). The possibility that some of the identified polyA sites are artifactual mRNA 3' ends due to internal priming was excluded by the absence of long genomic adenine stretches (> 6 bp) adjacent to the polyA cleavage site. Indeed, only the most represented polyA site (5th in Table 1) starts just downstream of an A_3_CA_4 _sequence (data not shown). Most polyA sites are associated with a polyA signal, defined as described in Methods, except for two cases (2nd and 6th polyA sites in Table 1). Moreover, the third polyA signal exhibits both unusual sequence and position compared to the polyA site (Table 1). The resulting alternative 3'UTRs range from 37 to 1018 bp, while the most common 3'UTR is 375 bp-long (5th in Table 1). The longest 3'UTR contains a cytoplasmic polyadenylation element (CPE), a signal known to be involved in the regulation of translational activation of quiescent maternal mRNAs during early development in animals [27].

All identified alternative 3'UTRs, together with the last 138 bp of the CDS of the *C. intestinalis *transcripts, are encoded by the last coding exon (61th exon).

No significant similarity to the *C. intestinalis *3'UTR was found in the *C. savignyi *genomic scaffold containing the htt CDS, nor in the remaining genomic sequences, preventing the prediction of *C. savignyi *3'UTR by similarity criteria. Moreover, there are no significant similarities between *Ciona *UTRs and the homologous regions of vertebrates [11,15,28,29] and *Drosophila melanogaster *[16].

As expected from the high level of polymorphism revealed by *C. intestinalis *genomic sequences [30], a number of positions with nucleotide differences were found in both the CDS and UTR of the sequenced transcript. In the CDS we identified a total of 76 polymorphic positions, with nucleotide differences resulting in synonymous (72%) and non-synonymous substitutions (28%, mostly conservative amino acid changes); in the UTRs both nucleotide substitutions and indels were found. Nucleotide differences were also observed between our sequences obtained by RT-PCR and data obtained by re-sequencing of publicly available EST clones (see Additional file Table S1), further confirming a high level of sequence polymorphism in this species. The observed pattern of nucleotide differences is in accordance with the evolutionary dynamics of coding and non-coding sequences, suggesting that such nucleotide differences are genuine polymorphisms.

### Protein analysis

**Figure 2:**
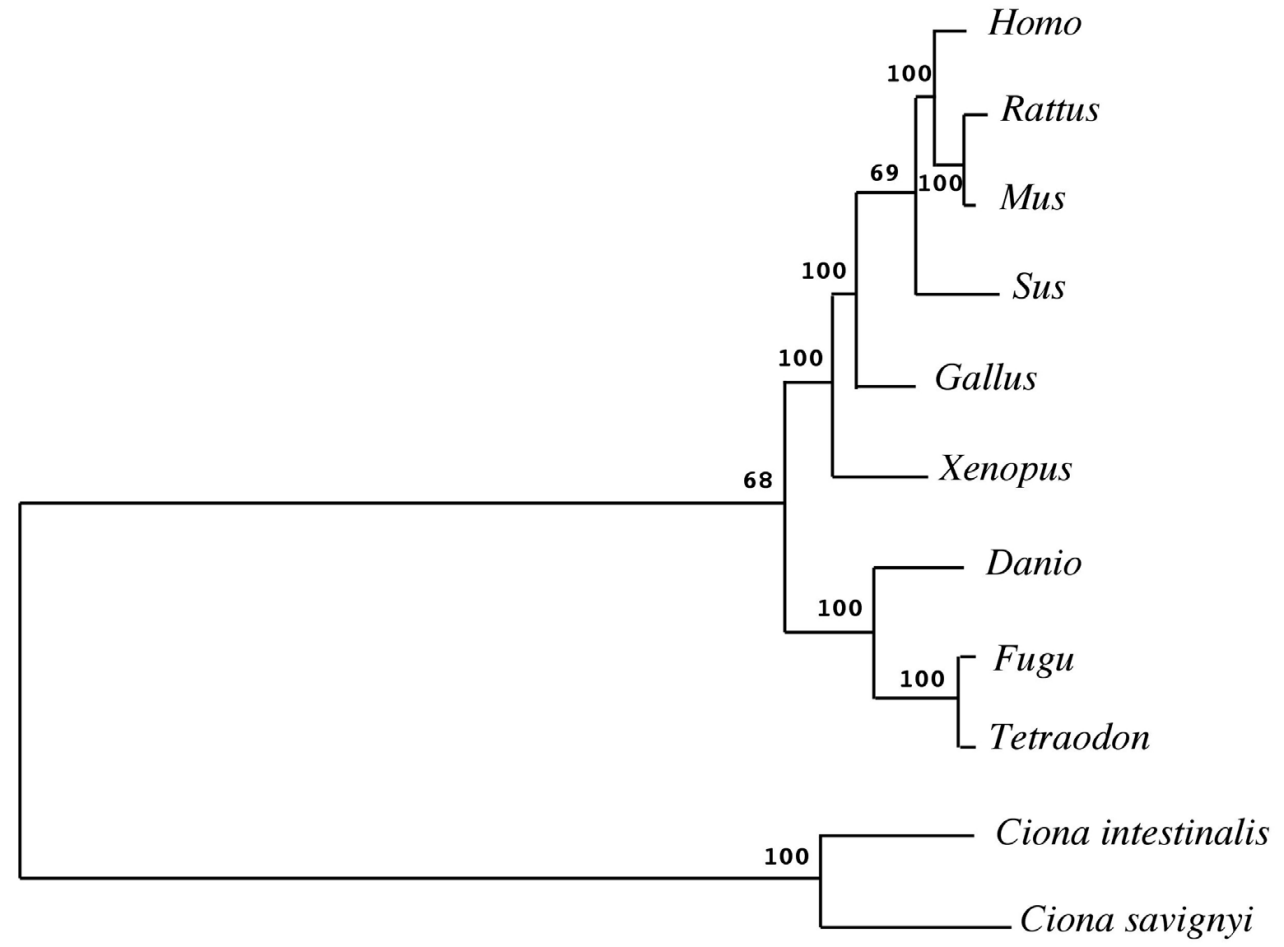
**Bayesian phylogenetic tree of huntingtin, reconstructed from protein sequences**. Branch lengths are proportional to the number of substitutions per site. Numbers close to the nodes represent Bayesian posterior probabilities.

The percentage of amino acid (aa) differences between all pairs of the eleven analysed chordate proteins are reported in Table 2. The sequence divergence between *C. intestinalis *and *C. savignyi *is as high as 27.95%, although these species belong to the same genus. This value is surprisingly high, particularly compared to the pairwise aa differences observed among vertebrates. Indeed, the divergence between pufferfishes belonging to the same family of Tetraodontidae (*Tetraodon *and *Fugu*) is only 4.97%, and that between mammals of the same order (mouse and rat) is as low as 2.86% (Table 2). On the contrary, the distance observed between the two *Ciona *species is comparable to that observed between mammals and fishes. The evolutionary tree based on amino acid data confirms the high divergence between the two *Ciona *species, and suggests the existence of long branches for these species (Figure 2).

The huntingtin proteins of *C. intestinalis *and *C. savignyi *are 2945 and 2946 aa long respectively, notably shorter than their vertebrate homologs, which are 3130 aa long on average. This length difference can be mostly ascribed to insertions/deletions in the N-terminal region of the protein (aa 1–1140 of the protein alignment, see Methods). Moreover, the N-terminal regions of both *Ciona *proteins lack the polyQ domain, or any kind of simple repeat (see Additional file 1). Even the proline-rich region typical of mammalian huntingtin is absent. A single histidine (*C. intestinalis*) or tyrosine (*C. savignyi*) residue is located at a position corresponding to the vertebrate polyQ stretch, suggesting low selective constraints acting on this region in the ascidian protein.

A total of 8 HEAT repeats (Table 3) are present in both the *C. intestinalis *and *C. savignyi *huntingtins. These are located as tandem arrays or as single elements in the N-terminal (4 repeats), central (2 repeats), and C-terminal (2 repeats) protein regions (each defined as one-third of the chordate protein alignment, see Methods). In human huntingtin, a total of 15 HEAT repeats are present, mostly clustered as tandem repeats in the N-terminal region (10 repeats, see Table 3). The analysis of htt protein alignment shows that all four ascidian HEAT repeats located in the N-terminal region and one repeat of the central region are also conserved in the same positions in the human homolog, whereas the HEAT repeats of the C-terminal region appear to be lineage-specific (Table 3). Thus, the presence of HEAT repeats at the N-terminal region seems to be an ancestral chordate character, further expanded in mammals. Finally, comparing the location of HEAT repeats to the gene exon boundaries, we found no indications that HEAT-repeats are encoded by single exons in either human or ascidians (data not shown). Thus, the protein domain structure does not correspond to the gene structure, at least with respect to the HEAT domains.

### Gene structure comparison

**Figure 3:**
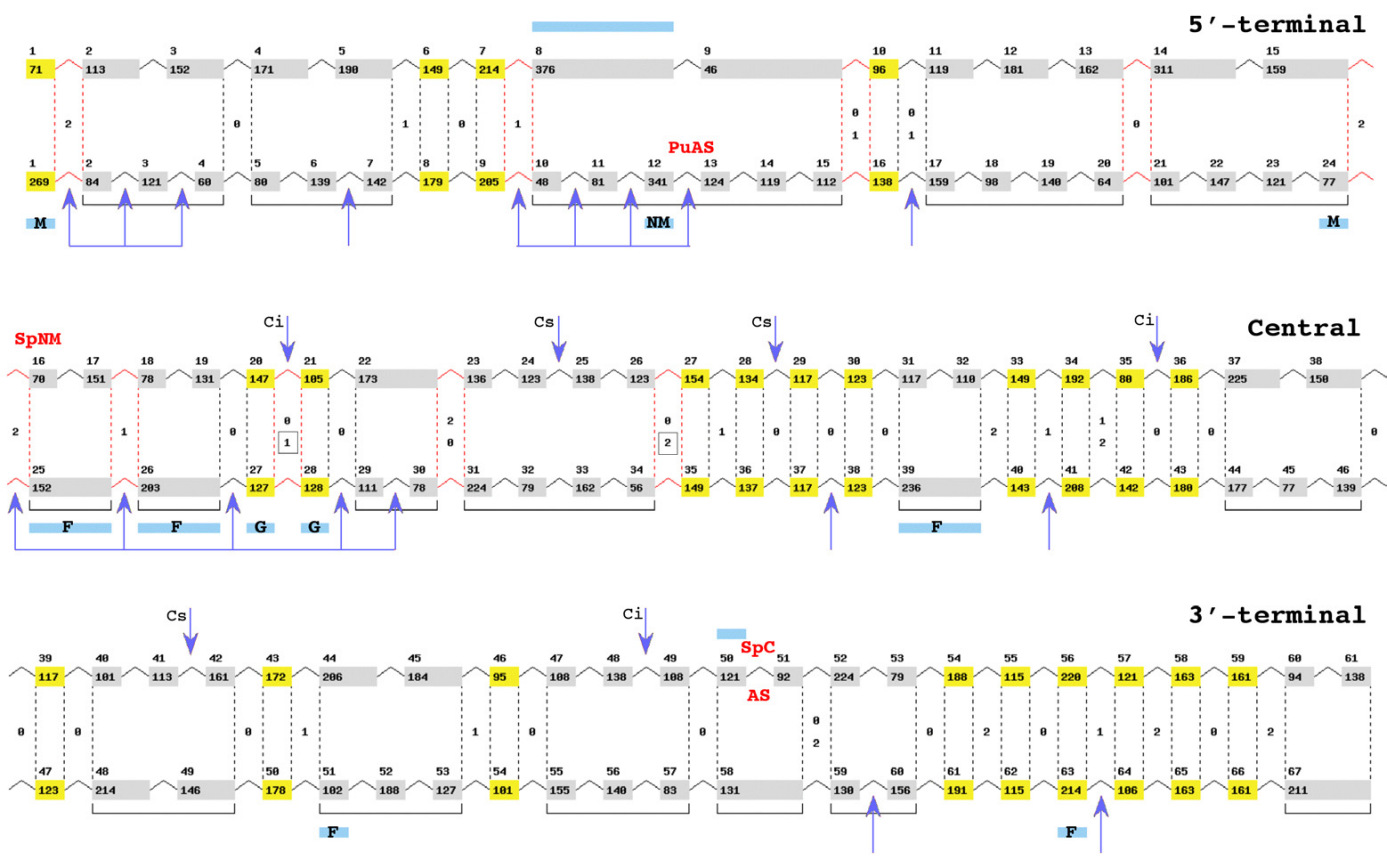
**Comparison of huntingtin gene structure between *Ciona intestinalis *(upper line) and *Homo sapiens *(lower line)**. Only protein-coding regions are indicated. Exons are represented by boxes, with upper numbers indicating exon numbering and inner number indicating exon length (in bp). Box size is unrelated to exon length. Square bracket: exon-block (see text). Yellow box: equivalent exon (see text). Gray box: exon belonging to an exon-block (see text). Introns positionally conserved in the two species are represented by dashed lines, in black for identical intron position, in red for slipped position (changes ≤ 18 bp). Intron phase is reported between dashed lines as a single number if common to the two species. Boxed phase number indicates that intron phase is not conserved in one vertebrate species (see text). Blue boxes below or above the gene structure indicate exons with length differences > 12 bp in vertebrates (below) or in the *Ciona *genus (above). Letters inside blue boxes indicates the species where the size difference is observed: M, difference between mammals and other-vertebrates; NM, difference within non-mammalian vertebrates; F, difference between fishes and other-vertebrates; G, difference only in *Gallus*. Arrows indicate the 5% longest introns in at least one vertebrate species (below), and in the *Ciona *genus (above). AS: alternative splicing experimentally identified in *C. intestinalis*. PuAS: putative alternative splicing identified "in silico" in *Gallus*, *Xenopus *and pufferfishes. Sp: presence of lineage-specific sequences only in non-mammalian species (SpNM) or only in *Ciona *(SpC).

The structure and size of chordate huntingtin genes (only CDS) are summarized in Table 4 and Figure 3. The coding region of *C. intestinalis *htt gene consists of 61 coding exons with length varying from 46 (exon 9) to 376 bp (exon 8) and a mean of 145 ± 55 bp (see Additional file 2). Introns account for 73% of the gene (Table 4). All introns are flanked by the canonical GT-AG consensus splice site and interrupt the coding sequence in all three possible phases. Phase 0 introns are the most abundant, with an approximate ratio of 3:1:1 for introns with phase 0, 1 and 2, respectively. The two alternative introns 50, due to the presence of a competing 5' splice site in exon 50 (Figure 1), share the same phase (1), and both follow the GT-AG rule. Such an alternative 5' splice site can be also predicted in *C. savignyi *(Figure 1), with the two alternative introns conserving phase 1 and canonical consensus splicing sites as in *C. intestinalis*.

The gene structure of the two *Ciona *species is almost identical (Figure 3 and Additional file 2): introns interrupt the CDS at identical amino acid positions and also in the same phase (only introns 34 and 41 have slipped six nucleotides). Similarly, orthologous exons are of consistently similar size, with substantial length differences (Δ L > 12 bp) found only in exon 8, and exon 50 (upper blue boxes in gene structure of Figure 3). Interestingly, exon 8 covers the less conserved region of ascidian huntingtin (57% local against a 72% overall amino acid similarity) and exon 50 is involved in the alternative splicing event.

The htt gene structure is perfectly conserved in the eight vertebrate species analysed, from fishes to amphibians and mammals, and consists of 67 exons (Table 4 and Figure 3). Introns are conserved in the same phase and identically positioned in all species, with few exceptions (intron 27 has slipped 19 bp in *Gallus*, and intron 34 has slipped 2 bp in *Tetraodon*. In both cases a phase change is observed compared to the remaining vertebrate genes). As in *Ciona*, phase 0 introns are the most abundant, with an approximate ratio of 2:1:1 for introns with phase 0, 1 and 2, respectively. Exon size is also highly conserved between vertebrates and ranges from 45–48 bp (exon 10) to 341–392 bp (exon 12), with a mean value of 140 ± 52 bp (see Additional file 2). Therefore, orthologous exons have almost identical size, and those with ΔL > 12 bp (lower blue boxes in gene structure of Figure 3) can be classified in three groups depending on the source of size variability:

• size differences between mammals and remaining vertebrates: exons 1 and 24 (indicated with M in Figure 3);

• size differences among non-mammalian vertebrates: exon 12 (indicated with NM in Figure 3);

• size differences between (one or more) fishes and remaining vertebrates: exons 25, 26, 39, 51 and 63 (indicated with F in Figure 3);

• intron sliding: the slippage of intron 27 in *Gallus *produces length variation of flanking exons in this species compared to other vertebrates but the overall amino acid length in this region is conserved (indicated with G in Figure 3).

In most cases, exon size variability reflects a propensity of the corresponding encoded protein region to accept multiple amino acid insertions/deletions: the extreme situation is found in exon 1, with the presence/absence and the length variability of the polyQ and polyP stretches. More interesting is the case of exon 24, which contains an additional 3'end portion encoding for 15–18 amino acids only in non-mammalian vertebrates. Similarly, length variation in exon 63 is found only in *Danio *and is due to 6 additional amino acids encoded by the 3'end of this exon.

Figure 3 reports the result of a comparison of the htt gene structure between ascidians and vertebrates, using *Ciona *and *Homo *as representative species. As many as 39 introns are positionally conserved in all chordates, exactly in the same position (30 introns, shown in black dashed lines in Figure 3), or slipped by at most 18 bp (9 introns, shown in red dashed lines in Figure 3). Moreover, 32 positionally conserved introns share the same phase (27 exact positioned introns, and 5 slipped introns). Thus, most shared chordate introns are conserved with respect to both position and phase, with a prevalence of phase 0 introns (ratio 18:8:6 for shared introns with phase 0, 1 and 2, respectively). The existence of common-chordate introns allows us to define:

• 23 "equivalent" exons, as orthologous exons whose sequence can be perfectly aligned over their entire length in all chordates (yellow exons in Figure 3);

• 17 exon-blocks, as groups of exons delimited by positionally conserved introns and containing lineage-specific introns, differently located in vertebrates compared to *Ciona *(grey exons grouped by square brackets in Figure 3).

Thus, the equivalent exons represent 38% and 34% of the total htt exon number of *Ciona *and vertebrates, respectively. Although the total number of exons is higher in vertebrates than in *Ciona*, there are 5 exon-blocks where one exon in vertebrates corresponds to two exons in *Ciona *but only one exon-block with the opposite pattern, suggesting that intron gains and losses have occurred in both lineages. The distribution of shared-chordate introns along the gene indicates that the 5'-terminal region of the huntingtin was more prone to taxon-specific intron gain/loss compared to the remaining regions. Indeed, there are only 8 shared-chordate introns in the 5'-terminal region, against 16 in the central region and 15 in the 3'-terminal region. Consequently, equivalent exons are quite limited in the 5'-terminal compared to the remaining regions of the gene (number of equivalent exons in the three regions 4:10:9) (see Figure 3).

The comparison of *Ciona *and vertebrate gene structure reveals the presence of two lineage-specific coding regions:

• in exon-block B_50_51, the *Ciona *sequence including the 3' end of exon 50 (long-isoform) plus the 5' end of exon 51 is absent in the central region of the orthologous vertebrate exon 58 (Figure 1), suggesting that the corresponding encoded protein region was lost by vertebrates or acquired by ascidians;

• exon 16 of *Ciona *encodes an amino acid sequence also present in non-mammalian vertebrates, where it is encoded by the end of exon 24 and the beginning of exon 25 (see Figure 3). Given its presence in *Ciona *and basal vertebrates, this sequence was most likely lost in mammals.

**Figure 4:**
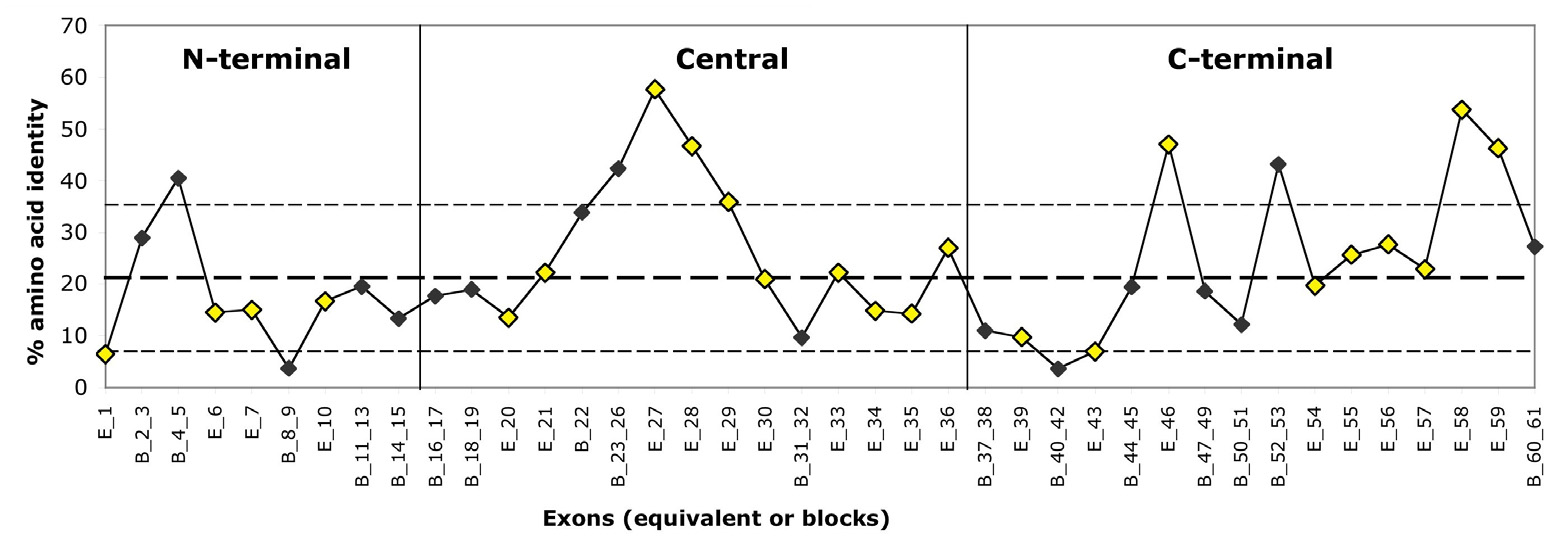
**Percentage amino acid identity calculated for each Equivalent exon (E, in yellow) and exon-Block (B, in gray)**. The percentage amino acid identity was calculated from the chordate protein alignment for each of the equivalent exons and exon-blocks described in Figure 1. Numbers refer to the *Ciona *exon numbering. Bold-dashed line represents the mean % identity (21.2%) calculated over the entire alignment length. Normal-dashed lines represent mean value +/- standard deviation (7.1).

The existence of a possible correlation between gene structure conservation and protein conservation was examined by calculating the percentage amino acid identity (% aa id) for each gene structural element shared by all chordates, that is equivalent exons and exon-blocks identified in Figure 3. Figure 4 reports the % aa identity for equivalent exons (E, in yellow) and exon-blocks (B, in gray) along the protein alignment, together with the mean % aa identity of the entire alignment (21.2 ± 7.1, bold dashed line in Figure 4). Equivalent exons are almost equally distributed above and below the mean % aa id, however the less conserved elements correspond to exon-blocks (B_8_9 and B_40_42), and the most conserved elements are equivalent exons (E_27, E_28, E_46, E_58 and E_59). Moreover, the most conserved equivalent exons belong to clusters of consecutive equivalent exons, indicative of a high conservation of gene structure, and are located in the central and C-terminal protein regions (Figure 4).

With regard to sequence conservation, a significant inverse correlation was found between % aa id and % gaps calculated separately for each gene structural element (R^2 ^= 0.416; data not shown), denoting that protein regions with a higher tendency to accumulate amino acid substitutions have also a higher tendency to accept insertions/deletions.

### Intron analysis

As shown in Table 5, intron length is highly variable within species. Generally, intron size does not correlate across species, and significant linear correlation between length of orthologous introns is found only in the species pairs *Fugu*-*Tetraodon *(R^2 ^= 0.65), *Xenopus*-*Gallus *(R^2 ^= 0.32) and within mammals (R^2^= 0.29–0.46) (values calculated excluding partial intron sequences).

The first intron is the longest intron in four of the eight vertebrate species (Table 5). Using a threshold to define unusually large introns (see Methods), the longest introns are mostly clustered in three regions of the vertebrate gene: two regions located at the 5' end of the gene (introns 1–3, and introns 9–12) and one region at the beginning of the central portion (introns 24–29, excluding intron 27) (arrows in Figure 3). In each of these regions, at least 6 of the 8 analysed vertebrate species contain an unusually large intron. On the contrary, there are no common gene regions where long introns cluster in both *Ciona *species (arrows indicated with "Ci" and "Cs" in Figure 3). Moreover, no correspondence between regions with longest introns in ascidians and vertebrates is observed (Figure 3).

**Figure 5:**
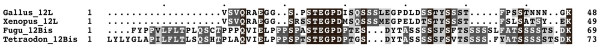
**Amino acid alignment of CSTs found in intron 12**. The Conserved Sequence Tag (CST) corresponds to an internal cassette exon (12Bis) in non-mammalian tetrapods and to a longest splicing isoform of exon 12 (12L) in pufferfishes. Identical, similar and conserved positions are indicated with different background.

A search for sequence similarities in intronic regions does not identify conserved sequence tags (CSTs) between the two *Ciona *species, nor between vertebrates and ascidians. On the contrary, intron 12 of vertebrates contains interesting CSTs in more than one species pair (Figure 5). Indeed, CSTs ranging from 102 to 177 bp were found in intron 12 in the species pairs *Xenopus*-*Gallus*, *Tetraodon*-*Fugu*, and *Mus*-*Rattus*. The *Xenopus*-*Gallus *CST has a high coding potential (CPS = 7.16) [31], whereas the *Tetraodon*-*Fugu *CST exhibits a marginal coding potential (CPS = 6.77), and the rodent CST is clearly non-coding, as indicated by a low CPS value (5.89). Moreover, the *Xenopus*-*Gallus *CST shows 34% amino acid sequence similarity to the CST of pufferfishes but no similarity to the rodent CST. The presence of a coding sequence in intron 12 is further confirmed by the prediction of a competing 5' splice site in exon 12 of *Xenopus *and *Gallus*, and of an internal cassette exon (that we called exon 12bis) in the two pufferfish species (SGP2 results), with all new putative introns following the GT-AG rule. This coding region, present only in some non-mammalian vertebrates and showing a low amino acid similarity (Figure 5), suggests the existence of an alternative splicing isoform due to a species-specific additional or longer exon located in intron 12.

### Gene size and repetitive elements

The variability of gene size between species is essentially due to changes in intron size (Table 4), which in turn correlate well with the overall nuclear genome size of the species considered. The total intron size increases proportionally with the overall percentage of repeated elements in introns (R^2 ^= 0.92) but a linear correlation between intron size and gene size still exists when intron length without repetitive elements is considered (R^2 ^= 0.95).

As shown in Table 4, repetitive elements cover a high and similar fraction of introns in all mammals, and a small fraction of the two pufferfish introns. The two species of *Ciona *show a similar fraction of repeated elements (9.8 – 12.5%), which is almost one-third of the mammalian repetitive intron fraction. A strong prevalence of interspersed is observed in all vertebrates, except for the pufferfish, whereas in the two ascidians interspersed repeats represent almost the same or more than twice the percentage of simple repeats (in *C. savignyi *and *C. intestinalis*, respectively) (Table 4). Among interspersed repeats, retroelements are more abundant than DNA elements in mammals and *C. savignyi*, whereas the opposite situation is observed in *Xenopus*, *Danio *and *C. intestinalis*.

Overall, the two *Ciona *species show a quite different distribution of repeats among classes, a situation rather surprising when compared with the similar repeat distribution observed between *Fugu *and *Tetraodon *and even between human and rodents.

No conservation of repetitive elements in orthologous introns in all or most analysed species is observed (data not shown). In *Ciona*, ten orthologous introns contain short low complexity repeats in both species (length < 70 bp), but there are no orthologous introns containing an interspersed repeat of the same class/family in both species.

## Discussion

Genomic data exclude the presence of paralogs of the huntingtin gene in the genus *Ciona*, as in both species the transcript sequence significantly matches only one genomic scaffold (or several scaffolds match different regions of the transcript, see Methods). The full-length huntingtin transcript of *C. intestinalis *shows several interesting features, such as SL trans-splicing, multiple polyadenylation sites, and alternative splicing involving the coding sequence.

The extent of SL trans-splicing has been recently investigated in *C. intestinalis*, revealing the esistence of polycistronic transcription units, and that about 50% of the total number of expressed genes are trans-spliced in this species [32]. A database search using the *C. intestinalis *SL sequence as a probe reveals that several full-length mRNAs and cDNA clones of *C. savignyi *also starts with the same SL sequence (data not shown). Considering that the trans-splicing status of individual genes is often evolutionary conserved in related species [25,33,34], we can speculate that the htt transcript is also SL trans-spliced in *C. savignyi *and in the larvacean *Oikopleura dioica*, a tunicate where about 12–24% of mRNAs are trans-spliced [35]. The SL trans-splicing found in *Ciona *htt could be responsible for specific mechanisms of post-transcriptional gene regulation different from those observed in vertebrates. Indeed, the 5'UTR of mammalian htt mRNA contains a conserved uORF that inhibits the translation of the downstream huntingtin ORF, at least in human [28]. uORFs are also present in the 5'UTR of the two *Ciona *species (only predicted in *C. savignyi*) but they are not conserved in either position or in sequence, so their functional significance is not obvious.

The htt transcript shows eight alternative polyA sites, mostly associated with polyA signals located at the expected position (10–30 nt upstream to the polyA tail), and with a sequence corresponding to one of the ten identified single-base variants of the AAUAAA vertebrate polyA signal [36] (Table 1). Moreover, the last polyA signal shows exactly the canonical AAUAAA sequence, in accordance with the observation that mRNAs with multiple polyA sites tend to use variant signals in the region proximal to the CDS, and a canonical AAUAAA at the 3'-most distal site [36]. The absence of and/or anomalies of three polyA signals can be ascribed to the existence of species-specific polyA signals, or to a radically different polyadenylation mechanism, as already suggested for human transcripts [36].

An alternative splicing event involving the CDS was fortuitously identified as being expressed in ovarian tissue. Since alternatively spliced isoforms were not specifically sought by our experimental strategy, we cannot exclude the existence of other transcript isoforms. EST data do not help in the identification of alternatively spliced isoforms but suggest an almost ubiquitous and low expression level of the htt gene in *C. intestinalis *(see Additional file 3), similar to observations in vertebrate and invertebrate species [16,17].

Overall, the maturation of the huntingtin transcript in this ancient chordate involves several processes, commonly reported as mechanisms of fine regulation of gene expression at the post-transcriptional level. Some of these processes are conserved even in vertebrates, and would indicate ancestral mechanisms of gene regulation. This is true for the alternative polyadenylation [11-14,29], and even for alternative splicing. Indeed, hints that alternative splicing occurs in vertebrate huntingtin may be gleaned from the literature [10,11], and our comparative analyses have highlighted the possible existence of a species-specific alternative splicing in some vertebrate species (Figure 5).

The exon-intron organization of the huntingtin gene is highly conserved in the two subphyla of Vertebrata and Tunicata. Previous studies on the exon-intron structure of small genes reported an almost invariant gene structure in vertebrates [37-40], and a hypervariability of intron position in the tunicate larvacean *Oikopleura *compared to both vertebrates and other tunicates, suggesting also a variability of gene structure within tunicates [40].

Here, we show that the exon-intron structure of a large gene, huntingtin, is almost identical between the two *Ciona *species and highly conserved between *Ciona *and vertebrates, with about 36% of the exons exactly conserved in both taxa. Assuming that introns in the same positions of orthologous genes are ancestral, the htt gene of the common ancestor of chordates should have contained at least the 39 introns positionally conserved in both *Ciona *and vertebrates (Figure 3). However, a more comprehensive reconstruction of the ancestral chordate gene structure will require the analysis of additional species, such as sea urchins, lancelet and other tunicates.

Splitting the gene into three regions, it appears that the 3'-terminal region was highly conserved in all chordates at level of gene structure, whereas the 5'-terminal was less conserved during chordate diversification (Figure 3), probably because of a relaxation or a modification of selective constraints. Indeed, the 5'-terminal region presents the lowest number of shared introns, and several slipped exons and intron phase changes, suggesting multiple events of intron gain/loss (Figure 3). In vertebrates, the longest introns are again concentrated in the 5'-terminal and the beginning of the central region of the gene, avoiding regions with high conservation of gene structure (Figure 3). Thus, we can hypothesize that the 3'-terminal gene region might have a low propensity to accept new sequences or intron expansions, because of the constraints to preserve specific functional elements. Finally, the 5'-terminal of the gene contains the less conserved exon-block (B_8_9), characterized by high variability at level of both structure (Figure 3) and sequence (Figure 4), and by the presence of a possible lineage-specific alternative splicing (Figure 5). Another lineage-specific sequence, lost in mammalian species, is located at the beginning of the central region (exon 16 of *Ciona*, corresponding to exons 24–25 of vertebrates), suggesting a trend to a lineage-specific evolution in the first portion of the vertebrate gene.

Introns are arranged independently of the predicted HEAT domains of the protein in both vertebrates and ascidians. According to the exon-shuffling theory [41], protein domains should correlate with the borders of their coding exons, particularly in protein categories functionally linked to organism multicellularity, such as extracellular and membrane proteins mediating cell-cell and cell-matrix interactions [42]. Although in huntingtin there is no indication of a correlation between the boundaries of HEAT domains and their coding exons, we can not exclude the existence of a correlation between the structure of the gene and the unknown domain structure of the protein.

As shown in Figure 4, only a weak correlation was found between sequence and gene structure conservation. This observation is in accordance with the result of a recent large-scale study on gene structure evolution in 11 deeply diverging animals species, showing that changes in exon-intron structure are gradual and largely, although not completely, independent of protein sequence evolution [43].

The htts of the two *Ciona *species show several striking differences: the amino acid divergence is similar to that observed between mammals and fishes (Table 2); the experimentally identified 5'- and 3'-UTRs of *C. intestinalis *did not allow the determination of the homologous UTRs *C. savignyi *by similarity criteria; a different distribution of repetitive elements among classes is found between the two species (Table 4); no orthologous introns contain interspersed repeats of the same class/family. Finally, there is no correlation between orthologous intron size of the two *Ciona *species, whereas a statistically significant correlation has been found between closely related species showing low CDS sequence divergence. All such data are in accordance with the considerable differences observed between the two *Ciona *species in the mitochondrial genome [44] and in nuclear coding and non-coding sequences [45], and suggest a high evolutionary rate or a ancient origin of the two *Ciona *species.

## Conclusion

Our comparative analyses of the huntingtin gene suggest that the 5'-terminal and the beginning of the central region of the gene were exposed to lower functional constrains during chordate evolution and underwent major changes at level of exon-intron organization, primary sequence, and intron size, with probable modification of the ancestral function or acquisition of new functions in vertebrates compared to ascidians. Thus, it is likely that the remaining central and 3'-terminal regions of the gene are those encoding for domains playing the ancestral gene function, shared by all chordates, and thus represent the ancestral traits of the huntingtin gene. In addition, the htt gene history suggests a high evolutionary rate of *Ciona *species compared to vertebrates and/or an early divergence time between the two *Ciona *species. These observations, derived from a single but highly informative gene, should stimulate further evolutionary studies of the *Ciona *genus.

## Methods

### Amplification and sequencing of huntingtin mRNA

A similarity search of the EST database by TBLASTN [46] identified 9 *Ciona intestinalis *EST clones significantly similar to the C-terminal region of human huntingtin (see Additional file 3). Moreover, one EST clone of *Ciona savignyi *matching to the N-terminal region of the protein was identified in the final stage of our work (Additional file 3). All clones were kindly provided by Dr. N. Satoh and Y. Satou, and were completely re-sequenced using universal and walking primers. In parallel, a first "in silico" gene prediction using the *C. intestinalis *genome assembly v1.0 (the one available at the time of the analyses), allowed the identification of five non-overlapping scaffolds partially containing the gene. All the sequence data obtained were used to define the experimental strategy to amplify and sequence the full-length cDNA sequence of *C. intestinalis*.

Total RNA was extracted from the ovary of two *C. intestinalis *individuals from the Stazione Zoologica of Naples, using the Trizol Reagent (Invitrogen) according to the manufacturer's protocol. After treatment of the isolated RNA with amplification grade DNase I, (Invitrogen), RT-PCR was carried out using the SuperScript™ III First Strand System (Invitrogen), and random hexamers to prime cDNA synthesis. Gene-specific primers, available on request, were all designed in different exons. The cDNA amplification strategy is schematically reported in Additional file 4: the transcript was amplified in seven fragments ranging in size from 1.1 kb to 1.9 kb, with overlapping sequences of at least 188 bp. Depending on yield and quality, the amplified fragments were directly sequenced after purification using Microcon-PCR Filter Centrifugal Devices (Amicon) or alternatively were cloned into pCR 2.1-TOPO vector using TOPO TA Cloning Kit (Invitrogen) and then sequenced (MWG Sequencing Service). 5' and 3' RACE were performed using the First Choice RLM-RACE kit (Ambion), following the manufacturer's protocol. In the 5' RACE random decamers were used for cDNA synthesis. An outer gene-specific reverse primer located in exon 5 and a nested primer located in exon 4 were used for amplification, obtaining a fragment of about 0.5 kb as product of the nested PCR. For the 3'RACE, the gene specific-outer primer (4 hF) was located in exon 53, and the inner primer (3 hF) was located in exon 54 obtaining multiple fragments ranging from about 1 to 1.8 kb. Products of 5' and 3'RACE were all cloned into pCR 2.1-TOPO vector using TOPO TA Cloning Kit (Invitrogen). Four positive 5'RACE clones and 23 positive 3'RACE clones were completely sequenced.

The *C. intestinalis *full-length mRNA sequence was deposited at the EMBL data bank under the accession number AM162277.

### Sequence analyses

The htt protein sequences of vertebrates extracted from Swiss-Prot, TrEMBL and REFSEQ databases (see Additional file 5) were refined using genomic data, and similarity criteria to experimentally well-characterized huntingtin proteins. In particular, protein sequences reported in the original database entry as "predicted" (such as those of *Gallus*, *Xenopus*, and *Tetraodon*) were carefully checked and extensively modified merging the initial sequence data to protein predictions obtained from GenScan [47,48] and GenomeScan [49,50] programs. The GenomeScan program was carried out incorporating information on htt proteins experimentally identified in closely related species (i.e. *Fugu *protein information was used for *Tetraodon *gene prediction). In addition, regions of original "predicted" entries with no significant similarity to other proteins or to ESTs of the same species (as defined by Blast analyses at [46]) were excluded from the final version of the protein prediction. The SGP2 program[51] was also used to optimize gene prediction in closely related species. SGP2 predicts genes by pairwise comparisons of genomic sequences, combining the tBlastx sequence similarity search and the Geneid "ab initio" gene prediction program [52].

The most recent genome assembly was used for the prediction of htt gene structure and protein sequence in vertebrates, except in the case of *Danio rerio*. For this species the genome assembly v4 was preferred to the up-to-date assembly v5 (May 2005), due to the presence in v5 of three genomic regions repeated in tandem in chromosome 1, each matching partially or entirely the experimentally determined transcript of this species [15]. On the contrary, in the genome assembly v4, only one genomic region, also located in chromosome 1, matches the huntingtin transcript of *Danio*.

All available genome assembly versions were used to reliably predict the htt gene structure and protein sequence in the two *Ciona *species (see Additional file 5), and an accurate comparison of the obtained results was carried out. The protein and gene structure prediction obtained from v1 and v2 assemblies of *C. savignyi *were identical, except for length and sequence of few introns. As regards *Ciona intestinalis*, in the assembly v2.0 the sequence of chromosome 5 matching the experimental htt transcript shows duplications and transpositions of some regions, whereas in the assembly v1.95 almost the entire htt transcript matches consistently the sequence of a single scaffold. Taking into account only the reliable region of the htt sequence identified in the v2.0 assembly, the gene structure obtained from the two assemblies of *C. intestinalis *was identical, except for the length of few introns and the lack of the last 400 nt of the longest 3'UTR in the v1.95 assembly.

In order to determine the exact exon-intron organization of the genes, the Gmap program [53,54] was used for mapping and aligning a given cDNA and/or coding sequence to the corresponding genomic sequence.

Protein alignment was performed with CLUSTAL W ver. 1.82 [55], and manually revised in an effort to maximize positional homology. The equivalent nucleotide (nt) alignment "back-aligned" from the protein data was also prepared (see Additional file 6). The chordate huntingtin alignment is 3420 amino acid (aa) long. In most analyses we considered the htt gene and protein split into three regions, each corresponding to one/third of the total alignment length, that is:

• N-terminal or 5'-terminal: protein alignment region 1–1140 (aa 1–839 and exons 1–15 of *C. intestinalis*, homologous to aa 1–1050 and exons 1–24 of human);

• central: protein alignment region 1141–2280 (aa 840–1882 and exons 16–38 of *C. intestinalis*, homologous to aa 1051–2107 and exons 25–46 of human);

• C-terminal or 3'-terminal: protein alignment region 2281–3420 (aa 1883–2946 and exons 39–61 of *C. intestinalis*, homologous to aa 2108–3144 and exons 47–67 of human).

To compare gene structure among species, exon sequences were mapped onto the nt alignment and the exact position and phase of each intron was verified by manual inspection.

The percentage of amino acid identity, the percentage of gaps and the uncorrected amino acid distances, corresponding to mean character differences per 100 aa adjusted for missing data, were calculated on the optimized protein alignment using PAUP* v4.0b10 [56].

The phylogenetic tree was reconstructed analysing the Gblocks-purified [57] huntingtin protein alignment (2148 sites, corresponding to 62% of the original positions), with the Bayesian method [58]. The JTT + gamma model was selected as best model fitting to the data, according to a ProtTest analysis [59] and following the Akaike information criterion. In the Bayesian analysis, one cold and three incrementally heated chains were run for 1,000,000 generations, with trees sampled every 100 generations from the last 900,000 generated (well after chain convergence).

Searches for protein domains were performed with InterProScan [60,61], and HEAT repeats were searched by the REP program [62,63].

Analyses of interspersed repetitive elements were performed using the RepeatMasker program ver. 3.1.2[64], selecting for each species the proper species-specific RepBase Update 10.4 library.

Intronic regions conserved across species (Conserved Sequence Tags, CST) were detected comparing all introns of a given species (previously masked from repetitive elements) against a database containing all intron sequences of the remaining species, through Blastn and Tblastx (E value < 0.01) [65]. CSTs shorter than 30 bp and/or identified only in orthologous introns of mammalian species or only in the species pair *Fugu*-*Tetraodon *were not further analysed. Assessment of the coding nature of detected CSTs was done through the computation of a coding potential score (CPS) on the total CST and on CST sliding windows and, using the CSTminer program [66,31]. CPS values higher than 7.71 indicate a coding potential of the related CST, and CPS values lower than 6.74 indicate noncoding CSTs, with an estimated false positive rate < 1% [31].

The 95th percentile of intron length of a given species was used as threshold to identify the longest introns of each species. Thus, introns scoring above the 95th percentile of a species were considered unusually long introns.

*In silico *search for experimentally-known regulatory elements in the 5' and 3' untranslate regions (UTR) of *Ciona *transcript was carried out using UTRScan [67,68].

The polyadenylation signals were searched in the 3'UTR sequence using the PatSearch program [69], and looking for a one-base variant of the canonical AAUAAA sequence in the 50 bp segment upstream each experimentally identified polyadenylation site.

## Authors' contributions

CG carried out the bioinformatic and molecular analyses, contributed to the study design, and drafted the manuscript. MT contributed to some bioinformatic analyses and helped to draft the manuscript. GP and EC conceived the study, supervised the research, and partecipated in its design. All authors have provided critical reviews of the manuscript content, read and approved the final manuscript.

## Supplementary Material

Additional file 1 Huntingtin amino acid alignment. Amino acid alignment, in fasta format, of the htt proteins of chordates. Click here for file

Additional file 2 Exon size of chordate huntingtin genes. Length (in bp) is reported only for proten-coding exons. Click here for file

Additional file 3 Huntingtin EST clones of *Ciona*. *Ciona *EST clones corresponding to partial huntingtin transcripts, and similarity to the htt mRNA sequence determined in this study Click here for file

Additional file 4 Schematic map of the *C. intestinalis *huntingtin transcript amplification strategy. Amplified fragments are reported as blue boxes; amplified and cloned fragments are reported as shaded boxes. EST clones, listed in Additional file 3, are reported as white boxes. Arrows indicate the position of inner RACE primers. The 5'- and 3'-UTR regions are shown as a thick line. Click here for file

Additional file 5 Protein sequence Accession Numbers (AC), genome assemblies and genomic coordinates of the region used in huntingtin annotation. Genomic coordinates refer to a sequence with about 10000 additional nucleotides upstream and downstream the region of initial similarity to the protein sequence Click here for file

Additional file 6 Huntingtin nucleotide alignment. Nucleotide alignment, in fasta format, of the htt protein-coding sequences of chordates. The alignment was obtained by"back-translation" of the amino acid alignment. Click here for file

## Figures and Tables

**Table 1:** Features of the huntingtin 3'UTRs of *Ciona intestinalis *mRNA and source supporting the data.

**N°**	**3'UTR (nt)**	**PolyA signal**		**N° clones/total**		**EST tissues**
		**Sequence**	**Position**	**3'RACE**	**EST**	

1	37	AAUAcA	-15	1/23		
2	118	-		1/23		
3	283	AAUcAA	-44	1/23		
4	323	AcUAAA	-15	2/23		
5	370	AAUAcA	-17	15/23	4/6	h, g, b, cl
6	496	-		0/23	2/6	eg, tb
7	744	AAgAAA	-18	2/23		
8	1018	AAUAAA	-10	1/23		

Lower case letters in the polyadenylation signal indicate differences from the canonical AAUAAA sequence. The position is the distance of the last base of the hexamer from the polyA start site, indicated as position 0. Tissue abbreviations: b: blood cells; cl: cleavage stage embryo; eg: egg; g: gonad; h: heart; tb: tailbud embryo. EST clones are detailed in Additional file 3.

**Table 2:** Uncorrected amino acid distances, calculated as average number of differences per 100 amino acids, for all pairwise comparisons of aligned proteins.

	**Species**	**2**	**3**	**4**	**5**	**6**	**7**	**8**	**9**	**10**	**11**
**1**	*Homo*	9.10	8.74	11.47	15.56	20.61	26.21	26.80	26.86	63.17	63.98
**2**	*Rattus*	-	2.86	13.05	17.21	21.78	27.26	27.96	28.19	63.31	63.93
**3**	*Mus*		-	12.75	17.07	21.74	27.16	27.64	27.87	63.40	63.95
**4**	*Sus*			-	18.46	23.75	27.90	28.19	28.23	63.15	63.56
**5**	*Gallus*				-	19.21	24.59	25.39	25.11	63.00	63.83
**6**	*Xenopus*					-	26.54	27.13	27.31	63.18	63.71
**7**	*Danio*						-	18.13	17.81	62.55	63.60
**8**	*Tetraodon*							-	4.97	63.64	64.23
**9**	*Fugu*								-	63.48	64.17
**10**	*C. intestinalis*									-	27.95
**11**	*C. savignyi*										-

**Table 3:** Ascidian and human HEAT repeats mapped on the protein sequence of the corresponding species.

**Species**	**HEAT name**	**REP E-value**	**Htt region**	**Location**	**Sequence**
*C. intestinalis*					
	A1	0.0005	N-term	58–96	PGLLAVSVETLLQSCADDNADVRLNANECLNRLIKGLYE
	A2	5.96E-06	N-term	139–177	RPYILNLLPCLCRISQREEDGVQETLGLSLVKIFKILGP
	A3	1.35E-06	N-term	181–219	ESEIQGLLASFLKNLSHKSATMRRTACVCLHSVILNCRK
	B4	6.19E-06	N-term	682–720	QSLSHQALSIALKCLCDDDLRLRKTAAATIVTMPTSFPT
	c	2.30E-06	Central	867–905	SQQQFGILPFVMSLLHSAWLPLDVTAHSDALVLAGNLVA
	E1	1.26E-06	Central	1341–1378	QGSASHVIPAMQPIIHDI.YVVRASSKNEPPEVTTQREV
	g1	9.05E-06	C-term	2771–2809	ARVMSKVLPSMLDDFFPAQDIMNKIIAEFISTLQPFPAS
	g2	1.46E-06	C-term	2864–2904	NRWISSMVPLIISRVHDPTLDVDWTCFCKAAVDFYTCQLSE
*C. savignyi*					
	A1	2.92E-07	N-term	58–96	PGLLAVSVETLLQSCADENADVRLNSNECLNRVIKGLYD
	A2	0.0001	N-term	139–177	RPYILNLLPCLCRISQREEDAVQEVLSSSLAKIFIVLGA
	A3	2.52E-06	N-term	181–219	ESEIQGLLASFLKNLSHKSPTVRRTACICLHSILTNSRK
	B4	1.53E-06	N-term	692–730	KSIAQKALSIALECLCDEDTRLRKTSSAAIVSMATSYPT
	c	1.46E-06	Central	876–914	AQQQFGILPIVMSLLRSAWLPLDVTAHSDALVLAGNLIA
	E1	-	Central	1352–1389	QGSASHVIPAMQPITHDI.FVVRGSLKNEPPEVTTQREV
	g1	1.27E-06	C-term	2770–2808	ARVMSKILPSMLDDFFPAQEIMNKIIAEFISTLQPFPGS
	g2	-	C-term	2864–2903	RWISSMVPLIISRSHDPSLDRNWTCFCKSAVDFYTCQLSE
*Homo sapiens*					
	A1	4.75E-07	N-term	124–162	QKLLGIAMELFLLCSDDAESDVRMVADECLNKVIKALMD
	A2	0.0001	N-term	205–243	RPYLVNLLPCLTRTSKRPEESVQETLAAAVPKIMASFGN
	A3	5.48E-07	N-term	247–285	DNEIKVLLKAFIANLKSSSPTIRRTAAGSAVSICQHSRR
	a4	*	N-term	291–329	SWLLNVLLGLLVPVEDEHSTLLILGVLLTLRYLVPLLQQ
	a5	7.77E-06	N-term	318–362	LTLRYLVPLLQQQVKDTSLKGSFGVTRKEMEVSPSAEQLVQVYEL
	b1	*	N-term	745–783	EYPEEQYVSDILNYIDHGDPQVRGATAILCGTLICSILS
	b2	1.04E-06	N-term	803–841	TFSLADCIPLLRKTLKDESSVTSKLACTAVRNCVMSLCS
	b3	*	N-term	842–880	SSYSELGLQLIIDVLTLRNSSYWLVRTELLETLAEIDFR
	B4	6.69E-08	N-term	904–942	KLQERVLNNVVIHLLGDEDPRVRHVAAASLIRLVPKLFY
	b5	9.05E-06	N-term	984–1025	RIYRGYNLLPSITDVTMENNLSRVIAAVSHELITSTTRALTF
	d	5.62E-06	Central	1425–1463	RLFEPLVIKALKQYTTTTCVQLQKQVLDLLAQLVQLRVN
	E1	*	Central	1534–1575	RKAVTHAIPALQPIVHDLFVLRGTNKADAGKELETQKEVVVS
	e2	*	Central	1610–1648	RQIADIILPMLAKQQMHIDSHEALGVLNTLFEILAPSSL
	e3	*	Central	1670–1710	TVQLWISGILAILRVLISQSTEDIVLSRIQELSFSPYLISC
	f	3.51E-06	C-term	2798–2836	DDTAKQLIPVISDYLLSNLKGIAHCVNIHSQQHVLVMCA

HEAT repeats are named according to their relative position along the chordate aligned sequences, using the same letter for repeats closer than 45 amino acids. Orthologous HEAT repeats conserved in ascidians and human share the same name, and are reported in upper case. The Expectation values (E-value) was calculated by the REP program [62]. Htt regions defined as in Methods. Absolute position of the HEAT repeats in the corresponding protein sequence is reported in the "Location" column. Dash: REP E-value not statistically significant. Asterisk: HEAT repeats originally described in Andrade and Bork [18] but not identified by the REP program as statistically significant [62].

**Table 4:** Chordata huntingtin gene structure, coding region (CDS), and length percentage of repetitive elements (Rpt) in intronic sequences.

		**Exon N°**	**Length (bp)**			**Intron L %**				
			**CDS**	**Gene**	**Introns**	**Rpt**	**Simple**	**Interspers**	**Retroel**	**DNA el**

Mammalia	*Homo sapiens*	67	9432	165202	155770	38.5	1.3	37.2	33.9	2.2
	*Mus musculus*	67	9357	146932	137575	38.2	1.9	36.2	34.7	0.5
	*Rattus norvegicus*	67	9351	145591 p	136240 p	35.4	2.6	32.9	31.3	0.9
Aves	*Gallus gallus*	67	9351	73424 p	64073 p	5.5	0.6	4.9	4.9	0
Amphibia	*Xenopus tropicalis*	67	9066 p	79087 p	70021 p	11.0	0.9	10.0	0	9.3
Teleostei	*Danio rerio*	67	9363	79017 p	69654 p	29.9	5.3	24.6	4.3	20.3
	*Fugu rubripes*	67	9444	21324	11880	1.1	1.1	0	0	0
	*Tetraodon nigridoviridis*	67	9435	22257	12822	2.4	1.8	0.5	0	0.5
Ascidiacea	*Ciona savignyi*	61	8835	45085	36249	9.8	2.7	7.1	4.4	2.1
	*Ciona intestinalis*	61	8838	32283	23445	12.5	5.8	6.7	1.6	5.1

Simple: satellites, simple repeats, low complexity repeats and small RNAs. Interspers: interspersed repeats. Retroel: retroelements. DNA el: DNA elements. p: partial sequence. Partial introns in *Rattus*: 1, 8, 28. Partial introns in *Gallus*: 46, 47. Partial or unknown exons in *Xenopus*: 27, 37, 66. Partial or unknown introns in *Xenopus*: 9, 26, 27, 36, 37, 65, 66. Partial introns in *Danio*: 1, 6, 8, 11, 13, 18, 25, 44, 49, 59. Intron 61 of *Danio *was excluded from gene size calculation due to its length (about 254 kb).

**Table 5:** Length variability of huntingtin introns.

	**Median**	**Average**	**Min**	**Max**	**First intron**	**95-th percentile**
*Homo sapiens*	1549	2360	93	12251	11850	9949
*Mus musculus*	1164	2084	92	20632	20632	6828
*Rattus norvegicus*	1146	2064	92	14532	14532	5970
*Gallus gallus*	743	971	85	5285	5285	2569
*Xenopus tropicalis*	831	1096	77	7186	7186	2849
*Danio rerio*	538	1091	73	4946	4891	3962
*Fugu rubripes*	105	180	66	1274	537	528
*Tetraodon nigridoviridis*	111	194	72	1264	781	736
*Ciona savignyi*	569	604	122	1933	331	1119
*Ciona intestinalis*	319	391	55	3038	257	616
