# Supplementary material for: Huntingtin gene evolution in Chordata and its peculiar features in the ascidian Ciona genus
Source: BMC Genomics. 2006 Nov 8;7:288. doi: 10.1186/1471-2164-7-288 (PMC1636649; doi:10.1186/1471-2164-7-288)
Supplement: Additional file 3 — Huntingtin EST clones of Ciona. Ciona EST clones corresponding to partial huntingtin transcripts, and similarity to the htt mRNA sequence determined in this study [file 1471-2164-7-288-S3.doc]

### Additional file 3

*Ciona* EST clones corresponding to partial huntingtin transcripts, and similarity to the *C. intestinalis* mRNA sequence determined in this study. EST clones were kindly provided by Dr. N. Satoh (for *C. intestinalis*) and Dr. Y. Satou (for *C. savignyi*)

| **Clone name** | **Tissue** | **5' EST** | **3' EST** | **L insert** | **Match to *C. intestinalis* htt mRNA** | | | | |
| --- | --- | --- | --- | --- | --- | --- | --- | --- | --- |
|  |  |  |  |  | L (bp) | **% Id** | **N° gap** | **CDS** | **3' UTR** |
| C. intestinalis |  |  |  |  |  |  |  |  |  |
| cibd055j13 | blood cells | BW044390 | BW016238 | 2146 | 2144 | 97.8 | 1 | x | x |
| cicl43e16 | cleavage stage | AV980994 | AV873327 | 1092 | 1090 | 97.3 | 1 | x | x |
| cigd038g02 | gonad | BW289146 | BW159675 | 1011 | 987 | 99.1 | 0 | x | x |
| cign050o02 | gastrula, neurula | BW268723 | - | 834 | 829 | 97.1 | 0 | x |  |
| cima035l20 | mature adult | BW484238 | BW493210 | 1090 | 1065 | 99.0 | 15 | x |  |
| citb31p16 | tailbud | BP018779 | AV877210 | 627 | 561 | 96.4 | 0 | x |  |
| citb10g19 | tailbud | AV674904 | AV679811 | 2009 | 1980 | 98.0 | 1 | x | x |
| ciht020l13 | heart | BW311719 | BW180886 | 589 | 579 | 96.0 | 1 | x | x |
| cieg099c12 | egg | BW220357 | BW085484 | 436 | 378 | 97.1 | 0 |  | x |
| C. savignyi |  |  |  |  |  |  |  |  |  |
| csga063k05 | - | BW521345 | - | 2986 | 2941 | 96.0 | 1 | x |  |
