# Supplementary figures and images for: Huntingtin gene evolution in Chordata and its peculiar features in the ascidian Ciona genus
Source: BMC Genomics. 2006 Nov 8;7:288. doi: 10.1186/1471-2164-7-288 (PMC1636649; doi:10.1186/1471-2164-7-288)

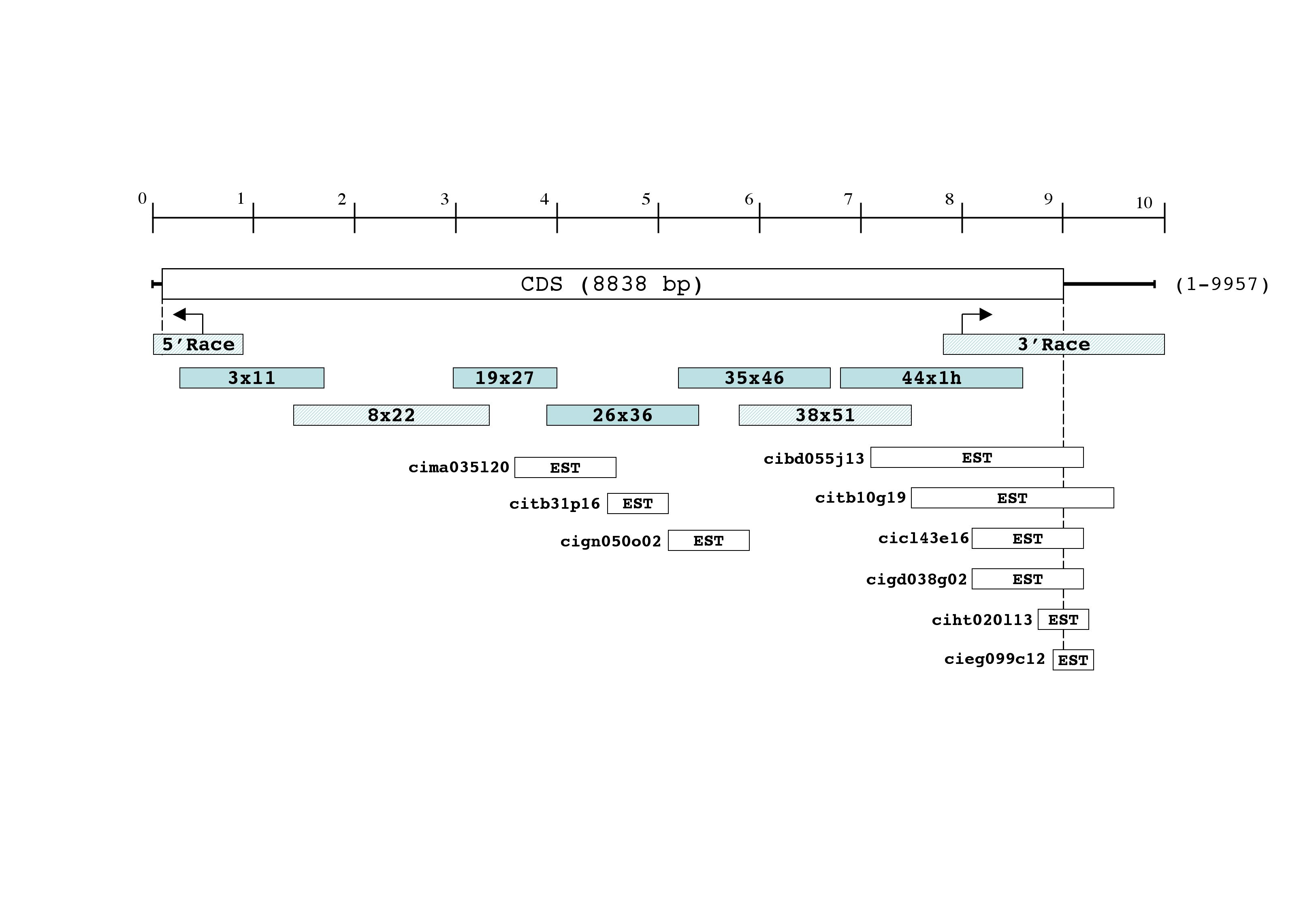

Supplement: Additional file 4 — Schematic map of the C. intestinalis huntingtin transcript amplification strategy. Amplified fragments are reported as blue boxes; amplified and cloned fragments are reported as shaded boxes. EST clones, listed in Additional file 3, are reported as white boxes. Arrows indicate the position of inner RACE primers. The 5'- and 3'-UTR regions are shown as a thick line. [file 1471-2164-7-288-S4.jpeg]
