# Supplementary material for: Huntingtin gene evolution in Chordata and its peculiar features in the ascidian Ciona genus
Source: BMC Genomics. 2006 Nov 8;7:288. doi: 10.1186/1471-2164-7-288 (PMC1636649; doi:10.1186/1471-2164-7-288)
Supplement: Additional file 5 — Protein sequence Accession Numbers (AC), genome assemblies and genomic coordinates of the region used in huntingtin annotation. Genomic coordinates refer to a sequence with about 10000 additional nucleotides upstream and downstream the region of initial similarity to the protein sequence [file 1471-2164-7-288-S5.doc]

**Additional file 5**

Protein sequence Accession Numbers (AC), genome assemblies and genomic region used for gene annotation (with additional 10000 bp upstream and downstream the initial region of sequence similarity to the protein).

|  |  | **Protein AC** | **Genome** | **Assemble** |  |
| --- | --- | --- | --- | --- | --- |
|  |  |  | **Date** | **Version** | Location (Strand) |
| Mammalia | Homo sapiens | P42858 | May 2004 | v35 | Chr4: 3103557-3288752 (+) |
|  | *Mus musculus* | P42859 | March 2005 | v34 | Chr5: 33239583-33406517 (+) |
|  | *Rattus norvegicus* | P51111 | December 2004 | v3.4 | Chr14: 81483481-81649080 (-) |
|  | *Sus scrofa* | BAA36752 |  |  |  |
| Aves | *Gallus gallus* | XP_420822 | March 2004 | WASHUC1 | Chr4: 82427283 – 82520706 (-) |
| Amphibia | *Xenopus tropicalis* | AAH80340 | October 2004 | v3 | Scaffold_458: 396979 – 496070 (+) |
| Teleostei | *Danio rerio* | AAC63983 | June 2004 | zv4 | Chr1: 44774097 – 45128028 (-) |
|  | *Fugu rubripes* | P51112 | August 2002 | v3.0 | ChrUn: 208584666 – 208625989 (-) |
|  | *Tetraodon nigridoviridis* | CAG03293 | February 2004 | v7 | Chr18: 4273940 – 4316191 (-) |
| Ascidiacea | *Ciona savignyi* | This study |  | v2 * | Reftig_48: 3151875 – 3216888 (-) |
|  |  |  | April 2005 | v1 | Scaffold_paired_23: 1046155 – 1111252 (-) |
|  | *Ciona intestinalis* | This study | October 2002 | v1.95 * | Scaffold_31: 333864 – 386142 (+) |
|  |  |  | March 2005 | v2.0 | Chr05q: 3722324 – 3788629 (-) |
|  |  |  | April 2002 | v1.0 # | Scaffold_54: 1 – 22208 (-)  §Scaffold_5386: 1 – 1282 (-)  §Scaffold_3429: 1 - 2171 (-)  §Scaffold_1561: 1 – 7654 (-)  ° Scaffold_540: 1 - 12850 (+) |

*: Assembly used in final huntingtin annotation.

#: Scaffolds matching to the huntingtin mRNA are listed from 5’ to 3’. A duplication in tandem of the first 2kb in present in scaffold_540.

§: The whole scaffold has been analysed
